# Disability-Related Questions for Administrative Datasets

**DOI:** 10.3390/ijerph17155435

**Published:** 2020-07-28

**Authors:** Rosamond H. Madden, Sue Lukersmith, Qingsheng Zhou, Melita Glasgow, Scott Johnston

**Affiliations:** 1 Centre for Disability Research and Policy, Centre for Disability Research and Policy, Sydney School of Health Sciences, Faculty of Medicine and Health, University of Sydney, Sydney 2006, Australia; Qingsheng.Zhou@health.nsw.gov.au; 2 Research School Population Health, Australian National University, Canberra 2601, Australia; 3 Western New South Wales Local Health District, Dubbo 2830, Australia; 4 Public Service Commission, New South Wales, Sydney 2001, Australia; Melita.Glasgow@dpmc.govt.nz (M.G.); scott.johnston@revenue.nsw.gov.au (S.J.); 5 Department of Prime Minister and Cabinet, Wellington 6004, New Zealand; 6 Revenue NSW, Parramatta 2150, Australia

**Keywords:** disability identification, disability statistics, workplace modification, inclusive employment, inclusive workplace, diverse workplace

## Abstract

High rates of unemployment among people with disability are long-standing and persistent problems worldwide. For public policy, estimates of prevalence and population profiles are required for designing support schemes such as Australia’s National Disability Insurance Scheme; for monitoring implementation of the United Nations Convention on Rights of Persons with Disabilities; and for monitoring service access, participation, and equity for people with disability in mainstream systems including employment. In the public sector, creating a succinct identifier for disability in administrative systems is a key challenge for public policy design and monitoring. This requires concise methods of identifying people with disability within systems, producing data comparable with population data to gauge accessibility and equity. We aimed to create disability-related questions of value to the purposes of an Australian state and contribute to literature on parsimonious and respectful disability identification for wider application. The research, completed in 2017, involved mapping and identification of key disability concepts for inclusion in new questions, focus groups to refine wording of new questions, and online surveys of employees evaluating two potential new question sets on the topic of disability and environment. Recommendations for new disability-related questions and possible new data collection processes are being considered and used by the leading state authority.

## 1. Introduction

Australia is among the countries recognising the rights of people with disability to participate fully in society and enjoy all the same opportunities, rights, and access to services as the rest of the community. In 2008, Australia ratified the United Nations Convention on the Rights of Persons with Disabilities (UNCRPD) [1], and now links major public policies and programs to UNCRPD objectives (e.g., National Disability Strategy, National Disability Insurance Scheme, and New South Wales (NSW) Disability Inclusion Act of 2014). Any success of these initiatives should be indicated by relative improvements in participation by people with disability in many life areas. The challenge for national statistics in any country is to produce reliable data capable of telling the participation story.

Data are therefore required on participation by people with disability compared to other citizens of the country, as well as on access to services, both specialist and mainstream. Relevant and efficient data collection requires concise methods of identifying people with disability within administrative systems, as well as the ability to compare the resulting data with population data in order to gauge accessibility and equity. Creating a succinct disability “identifier” for use in administrative systems is a key challenge for public policy design and monitoring in any country [2,3,4].

Meeting this challenge involves the design and adoption of short question sets, ideally a single question, in order to identify people with disability consistently within service data systems and monitor their access to, and experience of, generic services such as health and education, and to establish employment and other participatory experience in society [5]. A “disability flag” was designed by the Australian Institute of Health and Welfare (AIHW) to identify records of people with disability within data collections in various sectors. The flag aligns with Australian Bureau of Statistics (ABS) data about the need for assistance with self-care, mobility, or communication—a specific and less common level of disability [6].

The need for data development is illustrated by trends and data difficulties in important policy areas including employment and was recognised as a key need in the *World Report on Disability* [2]. Despite widespread disability policy reform in Australia and other countries, employment rates remain low, with Australia being the 21st of 29 OECD countries in 2010 ([7], pp. 49–51). Recent data indicate that employment of people with disability in Australia is not improving. Labour force participation and employment rates have decreased in recent years (Table 1).

The picture in the Australian state of New South Wales (NSW) public sector is no better, with the reported proportion of people with disability employed dropping from 4% in 2008 to 2.8% in 2016 (p. 32 and Figure 5.3 [9]). A target was set to double the representation of people with disability in the NSW public sector from an estimated 2.8% in 2016 to 5.6% by 2027 (in 2019 the disability employment target of 5.6% was announced as a Premier’s Priority for NSW and the end date to achieve the target amended to 2025) (p. 31, [9]). The NSW Public Service Commission (PSC) and the Department of Family and Community Services (FACS; now the NSW Department of Communities and Justice) are working to improve the inclusion of people with disability in the NSW public sector.

The decision of employees to report their disability status is complex; people with disability, when interviewed, cited reasons such as stigma, fear of discrimination, and irrelevance to work being performed in deciding not to share this information [10]. This issue could affect the quality of data.

The NSW PSC has a number of data collections designed to help it understand the needs of diverse employees, including people with disability:

- The Workforce Profile (WFP) is an annual census of the NSW Government workforce that has been collected since 1999. It captures a range of de-identified demographic information from employees of the NSW public sector that is extracted by government agencies from human resource (HR) systems. It collects information related to disability and environmental modifications—whether someone has indicated they have a disability, and whether they require an adjustment. In 2018, for example, representation of people with disability in the NSW public sector workforce was 2.5% [9].
- The People Matter Employee Survey (PMES) is a perception survey that encompasses the whole of the NSW government and is led by the PSC. While its focus is on understanding the engagement and experiences of its workforce, it also collects information on a range of demographic information and disability. It is an anonymous survey. Each year a higher proportion of respondents declare a disability than the WFP. In 2018, for example, 3.7% of survey respondents identified as having a disability [11].

This project aimed to create disability-related questions of value to the purposes of the NSW state PSC and contribute to the literature on parsimonious and respectful disability identification for wider application. Research was designed around the needs of the NSW PSC to inform recommendations on the wording of items in the WFP collection, to better (a) identify people with disability working in the public sector and (b) seek information on workplace modifications needed to promote full and effective participation in the workplace.

Specifically, the project reviewed the existing questions relevant to disability in the WFP and developed and tested new potential questions about disability (D questions) and the environment (E questions) to inform recommendations for changes to existing questions in the WFP, to

- Improve the response rate to disability-related questions.
- Improve the quality of data about disability and accessibility in the NSW public sector.
- Compare the NSW public sector workforce with the NSW population (proportion of disability according to Australian Bureau of Statistics data).
- Provide a better evidence base to inform policy interventions.

The detailed objectives of the project were to develop and trial disability-related questions and to assess how the questions were received and interpreted by NSW public sector employees, in order to ensure their appropriateness for use and inform change to questions in the WFP collection. The intention was to improve the quality of NSW public sector data on accessibility and disability representation in the WFP, assist in monitoring equity outcomes, and inform policy interventions to build a more inclusive and accessible workplace.

## 2. Materials and Methods

The project was undertaken in a partnership involving the NSW PSC, University of Sydney, and Department of Family and Community Services (FACS). The advisory sub-group (DEAC ASG) of the Disability Employment Advisory Committee (DEAC) comprises public sector employees with lived experience of disability; nominees were invited to join the project team. The DEAC itself consisted of disability peak body representatives, as well as experts from academia and the private sector. It advised the NSW government on improving the representation and inclusion of people with disability in the public sector. The DEAC was invited to comment over the course of the project. Four University of Sydney researchers, expert in the field, were project advisors.

The project was completed in three stages in 2017: (1) mapping and identification of key disability concepts for inclusion in new disability-related questions, (2) focus groups with NSW public sector employees to refine wording of new draft questions, and (3) online surveys to trial two potential new question sets with a wider group of employees. The University of Sydney gave ethical approval for the focus groups (no. 2017/132) and the online survey (no. 2017/254)—Stages 2 and 3.

Stage 1: Mapping and identification of key concepts

To identify key concepts for inclusion in disability-related questions, we examined 10 instruments of potential significance and relevance to disability data collection and measurement in the NSW public sector with reference to the International Classification of Functioning, Disability and Health (ICF) [12]. The method involved mapping key concepts, terms, and measures in each instrument to the ICF, enabling comparisons among the instruments by referencing a single framework. This approach is a well-accepted mapping or linking method for instrument examination and comparison in the disability field [13,14].

The ICF is the world standard framework and classification for organising information and data about functioning and disability. It defines the main components of functioning as body functions and structures, and activities and participation. It includes a classification of environmental factors to describe the physical, social, and attitudinal environment in which people live and conduct their lives. Environmental factors have a crucial effect (as facilitators or barriers) on people’s functioning and on the creation of disability in many life areas, including the workplace. The ICF represents a biopsychosocial model of disability, combining both the medical and social models of disability, thus recognising that disability may require both individual support and social, environmental change [15].

The ICF enables the collection and comparison of data relating to functioning and disability in many fields and is particularly suited to the present study; it provides a framework to underpin monitoring of implementation of the UNCRPD [4,12,16]. The World Health Organisation (WHO) has called for its wider use to increase worldwide disability data quality and consistency [2,17,18]. Australian statistical organisations such as the ABS and the AIHW adhere to such international standards in order to produce data that enables national and international comparisons and to capture consistent administrative data nationally [5,19].

Ten instruments were selected for mapping to the ICF, on the basis of their significance to the disability field, their relevance to disability data and measurement, and Australian population data that could potentially be compared with NSW public sector data. The selected instruments were the UNCRPD [1]; ABS Survey of Disability [20], Ageing and Carers (SDAC) [21]; ABS short module of disability for social surveys [22]; Household, Income and Labour Dynamics in Australia Survey (HILDA) [23]; Washington Group disability questions [24]; the current WFP questions [9]; and four other selected instruments, used primarily for research rather than population data collection [25,26,27,28] (Supplementary Table S1): Mapping instruments to the ICF).

Two authors carried out the mapping, one doing the initial mapping and the other cross-checking the results. Points of difference were discussed and resolved to reach consensus.

Stage 2: Focus groups to refine wording of new draft questions

The key concepts and terms identified in Stage 1 were used in different combinations, to draft five D questions (designed to identify employees with disability) and five E questions (designed to identify environmental factors that can facilitate, or act as barriers to, full and effective workplace participation); these questions were tested in focus groups with NSW public sector employees (Supplementary S1). The aim was to develop agreement within each focus group on (a) question clarity and user-friendliness, bearing in mind the project aims to improve data quality, comparability, and policy relevance, and (b) the questions and terms preferred by NSW public sector employees. The results guided the design of the questions to be tested in Stage 3 online surveys.

Of the possible focus group approaches, we used the nominal group technique [29,30]. This technique involved a structured approach to face-to-face discussion, with responses and ideas, as well as different iterations of the D and E questions which were discussed and clarified to develop consensus [31,32]. A facilitator encouraged all group members to participate and contribute. The nominal group was of a manageable size to enable discussion and agreement among participants.

The method and protocol for the focus groups were developed by the University of Sydney authors in collaboration with the PSC, and in consultation with members of both public sector departments and the DEAC ASG.

Five focus groups were held over an eight-day period in March–April 2017, in three locations (Sydney city, Parramatta, and Newcastle). An email invitation for focus group volunteers was forwarded by senior office holders and key contacts in the NSW public sector for distribution within their agency cluster (administrative arrangements that bring together different but related NSW public sector agencies), and DEAC ASG members and others with specific responsibility for diversity, including disability.

At least three of the authors were present at each focus group. A senior representative of the PSC involved in the project introduced the project and facilitators; a PSC note-taker was also present, with both PSC representatives joining discussions. Facilitators were University of Sydney project members.

Each focus group commenced with an attendee orientation to the purpose of the discussion, and “rules” were explained (e.g., mutual respect of opinions, opportunities to speak, confidentiality of the information shared, including any voluntary disclosure of personal circumstances such as disability). Facilitated discussions followed, about the draft D and E questions. Participants were asked probe questions such as “*Do you think the question is user friendly? What changes do you think should be made?*” The facilitators employed an iterative approach in group discussions using comments and opinions expressed by group participants to identify preferred concepts and those to exclude, then develop majority agreement on the terms and phrases preferred. All participants were encouraged to engage in the discussions. At the end of each focus group, a formal voting process invited participants to indicate anonymously their preference for two D and two E questions by placing an adhesive sticker on print-outs of all questions.

The analysis of the feedback from the focus groups involved synthesis of notes from all sessions. Links between question preferences and comments about preferred concepts, terms, and phrases were identified and discussed among all authors. Two new D questions and two pairs of new E questions were then developed by

- Using the key preferred concepts as the building blocks for questions, e.g., participation, environment, barrier.
- Reviewing focus group suggestions about the wording of questions and excluding or including particular terms and phrases.
- Using notes from the focus groups to inform the connecting words and phrases for the questions, e.g., “having” or “experiencing” difficulties or disability.

The D and E questions drafted after the focus groups were further refined after comment from project advisors from the University of Sydney and informal comments from key ABS staff. Two D and two pairs of E questions to be evaluated in Stage 3 online surveys were agreed after final discussion at a full team meeting (PSC, FACS, University of Sydney).

Stage 3: Online surveys to evaluate two draft question sets

In the final stage of the project, the two D questions and two pairs of E questions produced in Stage 2 were tested in two online surveys of a wider group of NSW public sector employees (Supplementary S2: online survey questions).

Criteria for judging the suitability of the questions in light of the project aims were developed to guide survey construction and analysis of the results. The relationship between each criterion and the survey—as well as other project elements—is summarised in Table 2. If the recommended D and E questions resulting from the research met all criteria, we would meet project objectives.

The survey instrument was constructed over several iterations, involving the whole project team and further consultation with DEAC ASG members. Two surveys were prepared to allow for presentation of test questions in a different order, and to avoid preferencing particular responses (Supplementary S2). At this stage, the question “*Do you have a disability?*” was added to the online surveys as Q11. This addition was in response to a concern expressed by some DEAC ASG members about the wording of the D questions (e.g., an apparent focus on impairment and health conditions, also present in WFP items). Online accessibility was checked by a specialist, leading to wording changes.

An email invitation to participate in the online survey was forwarded for distribution to the key disability contacts including the DEAC sub-group members, senior office holders for distribution to diversity employee networks (including disability) in their agency, and to employees more generally. The invitation emphasised the purpose of the project and specifically the online survey. The voluntary and anonymous nature of employee contributions was clarified in the invitation and participant information statement. People who responded that they were interested in participating were then sent one of the two surveys, according to their administrative cluster. A sample size goal of 200, for each questionnaire, was set in order to enable cross-tabulation of responses by broad demographic information. Surveys 1 and 2 were sent to respondents according to their cluster; that is, to approximately equal numbers of people based on cluster sizes of public sector agencies. In practice, this approach was modified, given the low responses to Survey 2 initially, and respondents after an extended deadline were provided with Survey 2.

## 3. Results

Results for the three stages of the project informed each following stage, resulting in one recommended D question and two pairs of recommended E questions for further consideration and development by the PSC (Box 3).

Stage 1: Mapping key concepts

The 10 instruments examined all had some concordance with the ICF. Accordingly, there was considerable overlap in key concepts and terms included in several instruments, indicating potential to produce comparable data. There was variation in emphasis placed on the three ICF components (body functions and structures, activities and participation, and environmental factors), and in the language and terms used to describe and capture data in these areas. The ABS SDAC was the most comprehensive of all the instruments examined, although it, like several others, was limited in its use of the environmental factors (see Supplementary Table S1 for further detail of these results).

Key concepts and terms most often included in the instruments are listed in Table 3, in three general categories broadly corresponding with three major ICF concepts.

The key concepts identified were used in various combinations to create five D and five E questions for discussion in the focus groups (Supplementary S1).

Stage 2: Focus groups

A total of 55 NSW public sector employees participated in five focus groups (32 females and 23 males), representing input from 15 agencies. Each focus group (9–16 people) ran for 2.5 h. No participant was asked to disclose whether they had lived experience of disability, although a number elected to do so; the group discussions reflected representation of the lived experience whereby participants disclosed at least 15 different disabilities and long-term health conditions amongst attendees. Given the aims of the study—namely, how best to elicit information to indicate disability—asking for such information in any form would have introduced circularity into the research.

Participant preferences on D and E questions, as voted at the end of each session, were tallied across the five groups. The most preferred D question was D4, followed by D3, and then D2; all these questions used key concepts or terms: “participation”, “impairments”, “long-term health conditions”, and “difficulties”. The preferred E question was E1, followed by E2, then E5; all these questions used key concepts or terms: “participate”, “equal”, “environment”, and “change”.

The implications about key words are difficult to interpret without the benefit of detailed comments on question structure, the format of lists used, and other expressions and terms. For example, the favourite D questions did not use the word “environment” and did use “not accommodated”, while the E questions (focused on the working environment) were preferred when they included “environment” and “enable” (E1 and E2) rather than “accommodate”.

Focus group members considered each question needed revision, with no D nor E question considered “final”. On completion of all focus groups, the project team drafted revisions on the basis of comments and opinions expressed by focus group participants (Box 1). The new questions thus developed included two D questions (D1 and D2) and four E questions (EN1 and EN2 relating to environmental barriers or changes needed now, and EP1 and EP2 relating to environmental facilitators or changes already made).

Box 1 **Sample comments made in focus groups.**

Use “work environment” rather than “workplace” as many people move around in their work (teachers, health professionals, transport workers).

Use a list rather than sentence for environmental factors.

Refer to “your environment” not “the environments”.

Terms such as “equality”, “on an equal basis”, or “equal opportunity” may be confused with diversity policies or imply comparisons with peers.

Mentioning “long-term health condition” is OK if used alongside “impairment”.

How to answer if you have an impairment but no everyday difficulty (because of management and environment)?

“Enable” a good word.

“Participate fully and effectively” discussed at length—and various alternatives offered.

The focus groups also began the process of obtaining comments on the draft introduction to the disability-related questions for use in the WFP, their likely effect on people’s responses, and the resulting data quality. Concerns were expressed about the use of data and its confidentiality, for example, concerns about potential disincentive to complete disability-related questions unless there was re-assurance on the use of data and its confidentiality, particularly regarding potential impacts on employment conditions and outcomes. Focus group participants advocated a simple explanation, which would give respondents answers to questions such as

- Why are you asking these questions, what are the data for?
- What is in it for me?
- What happens to the data (including who has access to it)?

These concerns were explored further in Stage 3 online surveys to inform implications for the introduction wording, question order, and discussion of the appropriate mechanism for collection.

Stage 3: Online surveys

Survey responses were received from a total of 533 NSW public sector employees (313 for Survey 1, 220 for Survey 2) from 41 agencies across 10 public sector clusters (27 agencies for Survey 1 and 20 for Survey 2, with 6 agencies represented in both surveys). Here, we summarise the results for D and E questions referring to survey questions by number (Supplementary Table S2). The percentages presented in this section are the percentages of valid cases, unless stated otherwise. All survey participants answered the questions, except for free text questions, resulting in the percentage of total cases being equal to the percentage of valid cases for most questions.

### 3.1. Results for the D Questions

Analysis of survey responses to Q1–Q11 indicated that both D questions (D1 and D2) tested are inclusive indicators of “disability”, and lead to the inclusion of more people than the question “do you have a disability?” (Q11):

- A higher percentage of people responded to D1 (26.2%) and D2 (30.5%) in a way that indicated disability than those who answered “yes” to Q11 (16.6% for Survey 1 and 26.8% for Survey 2) (Table 4 and Table 5, Supplementary Table S2). While disability in the NSW public sector, as reported on the basis of D1 and D2, was significantly more common than the current 3% reported in the WFP, no comparison can be made; people responding voluntarily to the online surveys are unlikely to be a random sample of NSW public sector employees.) The interpretation of disability in D1 and D2 is in line with the ICF world standard and the key concepts identified in the mapping exercise (Table 3). The results point to greater comfort answering D questions and their applicability to people who may not identify with disability, for example people with difficulties related to long-term health conditions (see data in Supplementary Table S2), and comments in Box 2).
- Both D1 and D2 “picked up” approximately 85% of people explicitly stating that they have a disability in response to Q11 (44 of 52 for D1, and 50 of 59 for D2) (Table 4 and Table 5).
- Significantly, D1 and D2, while showing a strong relationship with Q11, “captured” more people who were dealing with potential difficulties in their daily lives via technologies and other strategies.
- Of those who were not “picked up” by D1 (i.e., answered “no difficulty”), only 3.5% (8 in 231) stated later that they have a disability (in response to Q11) (for D2, only 5.9% (9 in 153)) (Table 4 and Table 5).

Box 2 **Inclusiveness of D1 illustrated by survey responses.**

I would answer yes to D1 and no to D2 because I have mobility issues due to arthritis but don’t consider I have a disability. But am impaired somewhat with mobility.

I technically have a disability (chronic pain and fatigue, mental health conditions) but have not disclosed this in past job applications. The questions being phrased in the more open way they are above would encourage me to respond more accurately.

Of the two D questions, it was concluded that D1 should serve as the basis for a final recommended D question for consideration and development by the NSW PSC. Descriptive statistics illustrated that (Table 6, Supplementary Table S2):

- Overall, D1 was considered slightly easier to understand than D2; 93% of respondents reported that D1 was “easy” to understand, compared to 91% for D2 (Q6); 12.5% felt that D1 had words that needed explanation, compared to 15.5% for D2 (Q7).
- Survey respondents were comparatively more “comfortable” answering D1 (in response to Q8)—there was little difference in Survey 1 responses; difference was more marked in Survey 2, with 63% in favour of D1 and 37% in favour of D2.
- The examples provided in D1 were appreciated by respondents to both surveys (Q9, Q10). Examples of everyday life areas were considered helpful by 68% of respondents to Survey 1 and 69% to Survey 2. Examples of long-term health conditions and impairments were considered helpful by 65% of respondents to Survey 1 and 60% to Survey 2.

The text comments offered throughout the survey help explain the statistics from the online surveys. These comments generally supported the conclusions of the statistical analysis, namely, to develop D1 into a recommended D question:

- “Impairment” was mentioned as a word needing explanation (21 people mentioned it in response to Q7, the most mentions of key words). There were few negative comments on impairment (two negative comments in response to Q7 made by people reporting disability in terms of D1 or Q11); the focus group discussions and the great majority of responses to the online survey did not flag any concern. Impairment is a term widely used in the disability field, including in the UNCRPD and ICF. Use of only “long-term health condition” in its place was not supported by focus group comments, which indicated a preference for using both terms; the examples of “conditions and impairments” listed in D1 (favoured by survey respondents) do contain impairments. Use of impairment categories (physical, mental, intellectual, sensory) from D2 was not recommended; a number of text comments from the survey sought explanation of the bracketed words.
- The use of the word “disability” in the questions appears to exclude some people from responding, e.g., those with functioning difficulties arising from long term health conditions; this is the balance of evidence from the online survey statistics, text responses, and focus group results. “Disability” would perhaps better be used in the introduction to the question.
- The word “participation” was well received by participants in focus groups and in the online survey; it was not included in D1 in the survey but should be in further development of D1.

Both privacy and use of the data appeared significant concerns in text responses to the introduction and requests for “any other comments”, reinforcing focus group views. People reported lacking knowledge of and confidence in how data are used.

### 3.2. Results for the E Questions

On the basis of analysis of survey responses to Q13–Q21, we concluded that, of the four E questions (EN1 and EN2 relating to environmental barriers to functioning or changes needed now, and EP1 and EP2 relating to environmental facilitators of functioning or changes already made), EN1 and EP1 should serve as the basis for two final recommended E questions for consideration and further development by the PSC (Table 7, Supplementary Table S3):

- The great majority of survey respondents (95% or more) found all four E questions “very easy”, “easy”, or “somewhat easy” to understand (in response to Q14 and Q18). A correspondingly high proportion said no further words needed explanation (e.g., 93% for EN1 and 89% for EN2 (Q15)). A higher proportion of respondents indicated that words needed explanation in EN2 (11.4%) and EP2 (10.3%) compared to EN1 (7.1%) and EP1 (6.0%) (in response to Q15 and Q20) (Supplementary Table S3).
- Respondents demonstrated no difficulty in responding across all five areas of the ICF environmental factors, and in differentiating present needs and needs met. The questions have the capacity to yield interesting information (Supplementary Table S3).
- There was greater comfort answering EN1 than EN2 (in response to Q16)—59% of people in Survey 1 were more comfortable answering EN1; preferences were evenly split in Survey 2, with 51% preferring EN1 and 49% preferring EN2. The word “improvement” (in EN1) appeared to be favoured over “adjustment” (in EN2). Similarly, EP1 was preferred to EP2 (in response to Q21).
- A higher percentage of people indicated that “improvements” are needed in all five aspects of the environment in response to EN1 compared with those who indicated that “adjustments” are needed in response to EN2 (Q13). Correspondingly, a lower percentage of people indicated “no improvement needed” (40.6%) in response to EN1 compared with those who indicated “no adjustment needed” (56.4%) in response to EN2. A similar but less marked pattern was seen for EP1 and EP2 (Q.18)—57% in Survey 1 reported no past improvements, and 65% in Survey 2 reported no past adjustments. The results could reflect a difference between the samples, or that EN1 and EP1 are open to wider interpretation.

The relatively small number of text comments about E questions (24 in each survey) generally supported the conclusion of the statistical analysis—namely, to develop EN1 and EP1 into two recommended E questions, with attention to suggestions for improvement. Free text comments from survey respondents, with feedback from focus groups, provided guidance for amending the questions, and could also be used to improve layout:

- Generally, people preferred simpler wording, without jargon and need for further explanation. Comments (in response to Q15 and Q20) reinforced preferences for the term “improvement” rather than “adjustment”, which was frequently mentioned as a word needing explanation. Other problematic terms reported include “human-made”, “enable”, and “colleagues”.
- There was a suggestion from Survey 1 to simplify “what aspects of your work environment” to “at work” ... “enable you to participate fully and effectively”.
- From Survey 2, suggestions included the need for further explanation or alternative terms such as “adjustments”, “EN2 asks me what help I need”, “EN1 feels like it is seeking a criticism about my workplace”, “EN2 reads like we all need adjustments, and ‘what adjustments have you and your employer made’ may be more positive”.

Overall, the free text responses about both the D and E questions, briefly illustrated in this paper, provided rich material for question redevelopment.

## 4. Discussion

With the aim of improving the WFP questions and data, the research developed a new set of questions (Box 3) related to disability and accessibility, comprising one D question to identify disability, and two E questions about environmental modifications. The E questions enable data to be captured on improvements already made, as well as improvements that need to be made in the future. The mapping stage of the project ensured that the new questions correspond conceptually with the UNCRPD, the ICF as the world statistical standard, and ABS data.

The new questions performed well against the objectives of the project:

- **Questions are inclusive**: The new questions bring into scope more people than the question “do you have a disability?”—for example, people with long-term health conditions who may not identify with disability, as well as people using technologies and other strategies to manage difficulties in their daily life.
- **Comparable to NSW population data**: The new questions use key concepts used in other data collection instruments (as well as key policy instruments), thus promoting comparability with the NSW population and labour force data.
- **Clarity and meaning**: The new questions were preferred to the alternatives as they were considered easier to understand.
- **Comfort in answering**: The new questions were preferred to the alternatives as more employees were comfortable answering them.

### 4.1. Recommended D and E Questions

Following all three stages of the research, including analysis of statistical and text results of the online survey, which informed further refinement of the questions, Box 3 sets out the recommended D and E questions. The inclusion of “participation” should be tested in any further refinement of the D question; while D1 was the preferred question, it did not include this popular word. Box 3 illustrates how it could be included.

There was strong support for lists of examples: of health conditions and impairments that may interact with the environment to create the experience of disability, as well as for areas of the environment involved in this interaction. Example lists used were based (respectively) on the list used by the ABS in the SDAC and the environmental factors classification of the ICF; the environmental lists and examples were seen as easy to understand and to require little explanation.

Box 3 **Recommended D and E questions.**

**Question D1:**

In your everyday life, do you experience difficulty (**participating)**, related to a long-term health condition or impairment, in any of the following:

Social and community life (e.g., recreation, sport, religion).
Work, education or training (including paid or voluntary work).
Mobility (e.g., walking, moving around, handling or lifting objects, using public transport).
Self-care (e.g., washing, dressing).
Home life (e.g., shopping, cooking, caring for others).
Daily organisation (e.g., undertaking multiple tasks, making decisions, handling stress).
Communication (e.g., speaking or using communication devices).
Learning (e.g., basic learning, or applying knowledge in solving problems or making decisions).
Relationships (e.g., with friends, family, supervisors, co-workers or acquaintances).

Please select one of the following:

- Yes, I sometimes or always experience difficulty in at least one area, even when I use equipment, technology, assistance, or other techniques.
- No, but I use equipment, technology, assistance, or other techniques to avoid difficulty.
- No difficulty.

Note: Long-term health condition or impairment may refer to the following:

Loss of sight or hearing;

Speech difficulties;

Shortness of breath or difficulty breathing;

Ongoing or repeated pain or discomfort;

Blackouts, seizures, or loss of consciousness;

Difficulties learning or understanding things;

Difficulties using arms or fingers;

Difficulties using feet or legs;

Mental health conditions;

Restricted in physical activities or in doing physical work;

Memory problems;

Social or behavioural difficulties;

Head injury, stroke, or other acquired brain injury;

Or any other long-term conditions.

 

**Question EN1:**

Do aspects of your work environment need to be improved to enable you to participate fully and effectively at work? (Select all areas where improvement is needed):

Support and relationships (e.g., support from co-workers or supervisors).
Attitudes (e.g., attitudes or behaviour of colleagues, supervisors, or clients).
Services, systems, and policies (e.g., job design, working hours, flexible work options, transport, employment policy, training, or workplace and hiring policies).
Products and technology (e.g., work equipment, ICT, furniture, building design, and access).
Natural environment and human-made changes to environment (e.g., noise, light, air, or water)

No improvements needed.

EP1 similar to EN1 but referring to the past i.e., what has been done:

Have aspects of your work environment been improved to enable you to participate fully and effectively at work?

### 4.2. Future Research

Further analysis of text responses to the online survey and of notes taken during focus group discussions could lead to even further refinement of words and examples in D, along with further plain English editing.

Further discussions could be undertaken with agencies with interest in disability data, including the Australian Human Rights Commission. Further testing of the questions in another English-speaking country with similar national data, or use of the process described here to develop relevant national questions, may provide useful insights and comparisons in similar work-related contexts.

The strong association between the new disability questions, about difficulties participating in everyday life, and the question “Do you have a disability?” (Q11), is interesting and perhaps reassuring. Nevertheless, some analysis suggests areas of future research. Notably, D1 and D2, while showing strong relationships with Q11, “captured” more people who were dealing with potential difficulties in their daily life using technologies and other strategies. This finding reflects views expressed in the focus groups that the existing D question in the WFP (similar to Q11) may lead to self-exclusion. For example, as illustrated by the focus group and survey comments, people with difficulties related to long term health conditions or people whose difficulties are mitigated by technology or environmental facilitators may have varying views of disability and may not identify or declare they have a disability.

The apparently greater inclusivity of the new questions resonates with results from related research in a vastly different setting. Disability identification in refugee situations, for the purposes of offering services and determining and meeting needs, was found to be best done using “functionality-based questions in line with international standards” along with staff training to enable “greater sensitivity to the many different ways in which disability can manifest” ([33], p. 60).

## 5. Communication

Communication at various points will be an essential part of improving data. Communication may

- have various purposes and messages—communication about system changes; reminders about the need for updating data, the value of the data, and of participation in providing it; explanation of the importance and purpose of the questions; and accurate assurances about privacy and confidentiality; and
- use various avenues—announcements, publications, presentations, and routine communication, e.g., in forms and databases including through explanation of and introduction to the questions.

A communication strategy could be devised on the basis of the findings of this project and decisions made about implementation in order to support the chosen paths forward on data collections relating to disability in the NSW public sector.

### The Purpose, Use, and Place of the D and E Questions Require Discussion

Further high-level consideration by the PSC about the purpose and use of the disability (D) and environment (E) questions is essential in order to establish the basic “why, who, when” purposes and processes. There was strong comment in both the focus groups and online survey, revealing distrust among NSW public sector employees concerning the purpose of the D question in particular and about data confidentiality.

The most appropriate data collection vehicle to gather information on disability and accessibility requires consideration. Some Australian government jurisdictions collect their disability data through HR systems and others through sample employee surveys (anonymous and confidential), possibly similar to other countries. There are advantages and disadvantages to both methods (Supplementary Table S4).

The PMES is anonymised and online, and not linked to HR systems. The PSC has control of the questions and is the sole data custodian. However, because the recommended questions are long (relative to others in that survey), so as to reflect the complexity of disability, there are advantages to using them in a context where this complexity can be explored. Therefore, the WFP collection, which draws information from HR systems, may remain the better approach, as it allows the accumulation of more in-depth data over time; the WFP collection was the focus intended at the start of this research. The questions could be asked during HR processes and stored in HR systems; if so, the purpose needs to be clear and assurances about use and privacy provided and demonstrated. While the purposes may include monitoring disability employment rates, locating the data with HR creates the capacity to cross-tabulate disability data with other relevant HR data (e.g., with position/level, age, and gender) and statistically monitor change over time.

Trust and comfort in reporting may also be influenced by the inclusiveness of the workplace, where employees feel “safe” to share their personal information. Both elements—technical and cultural—are important in developing a better picture of workplace inclusion of people with disability—having appropriate survey questions, ethical data storage and use practices, and an environment where employees feel their information is respected and acted on constructively.

The links between the D and E questions also need to be decided. It has been assumed that the D question comes first and acts as a filter to direct people to the E questions. An alternative could be to use the E question as the first question, and then ask for explanation of why environmental change is needed, even perhaps using all “diversity” questions as possible reasons. This approach has not yet been tested and has policy implications. In favour of this re-ordering is the finding that the E questions did not elicit the types of free-text comments made about the D questions. It was clearer why E questions were being asked and what actions might be taken on the basis of answers. However, there remains a question about timing; for example, people may find it hard to comment soon after recruitment on what environmental improvements they need.

The results of this project thus raise wider questions of data collection strategy. One option is to re-think the use of two questions, on disability and environment, as part of an overall strategy to improve understanding of disability and changes needed in the workforce environment. The questions could be used to monitor changes in the environment as well as in the representation of people with disability in the workforce.

The OECD noted, in contrasting disability policy reform with the lack of improvement in employment rates, “disability system reform is a huge task, for several reasons” (p. 93 [7]). Many months after completion of this research, the discussion about next steps is still in play. The research has been used to inform enhancements to the PMES and shared with other jurisdictions. Inevitably, key senior staff at the PSC have moved on during this period (including Johnston and Glasgow) with the resultant need for handover communication. In addition, the NSW government announced the Jobs for People with Disability Plan, which has resulted in a significant reprioritisation of work to advance disability inclusion across the NSW public sector workforce. Improving the accuracy and reliability of data remains a key focus area. Inclusion of a clear definition of disability was drafted using this research and is to be implemented into the WFP to provide more clarity of what is included. While it is understood that the WFP remains the most appropriate vehicle for the collection of detailed data public sector-wide, implementing change to the WFP is nevertheless a significant challenge—it affects administrations across the NSW public sector (with the NSW government being the largest public sector employer in Australasia). The strategic timing of such a change is made more complex by the impact on public administration in 2019–2020 of a long-term drought, severe bushfires, and now the COVID-19 virus.

## 6. Conclusions

In the public sector, creating a succinct identifier for disability in administrative systems is a key challenge for public policy design and monitoring. This requires concise methods of identifying people with disability within systems, producing data comparable with population data to gauge accessibility and equity. This paper focussed on efforts to improve data in the service of evidence-based policy. The project described here formed part of a broader program of work to improve interventions that build inclusive work environments and enable people with disability to have positive work experiences in and contribute to the NSW public sector.

We aimed to create disability-related questions of value to the purposes of an Australian state and contribute to literature on parsimonious and respectful disability identification for wider application. Both these aims were achieved. Recommendations for new disability-related questions and possible new data collection and related communication processes are under active consideration by the leading state authority, as are the additional factors identified during the collaborative research relating to the design of and communications about administrative data collections. More broadly, this research contributes methodology and findings relevant not only in the Australian public sector, but in other countries with good quality national population data with which administrative data can be compared.

## Figures and Tables

**Table 1:** Trends in labour force participation and employment of people with disability in Australia (15–64 years).

-	2009			2012			2015		
Percentage of people with disability who are in the labour force	54.3	±	1.1	52.8	±	1.3	53.4	±	1.5
Percentage of people with disability who are employed	50.0	±	1.0	47.7	±	1.3	48.1	±	1.6

Source: Table 15A.73 and 5A.74 [8].

**Table 2:** Project elements to ensure project criteria met.

Criterion for D and E Questions	Survey or Other Study Elements
Improved response rate to disability questions (requiring clarity, meaningfulness, and user-friendliness) based on broad categorisations preserving non-identifiability of individuals.	Clarity—Q6 (how easy is question D1 to understand?). Meaning—Q7 (do words need explanation); and Q9 and Q10 about preference for examples of life areas and health conditions. User-friendliness—Q8 (comfort answering).
Improved data quality for disability in NSW public sector (validity in terms of alignment with key current disability concepts).	Mapping to instruments (Stage 1).
Ability to compare NSW public sector workforce with NSW population (ABS data) (requiring use of similar concepts to those of the ABS SDAC).	Mapping to ABS data concepts (Stage 1), informal consultation with ABS (Stage 2), and question design (Stage 3).
Better evidence base to inform policy interventions (requires personal and policy relevance of the questions).	Mapping (Stage 1), focus groups (Stage 2), online survey (Stage 3).

**Table 3:** Key concepts of disability that support data comparability, for use in surveys.

Components of Functioning	Environment and Effects	Disability Being Related to “Health Condition”
Participation Activities Impairment Difficulty with Activities	Environment Interaction Barrier in environment, hindering participation Difficulty with participation in environment … on an equal basis	Long-term health condition Impairment Restriction

Underline in table is to emphasize the meaning.

**Table 4:** Responses to D1 and Q11.

	Question D1 on Difficulty in Listed Areas of Life			
Question 11: Do you have a disability?	“I sometimes or always experience difficulty in at least one area, even if…”	“No, but I use equipment, technology, assistance or other techniques to avoid difficulty”	No difficulty	Total
Yes	32	12	8	52
No	13	25	223	261
Total	45	37	231	313

**Table 5:** Responses to D2 and Q11.

	Question D2 on Difficulty in Listed Areas of Life			
Question 11: Do you have a disability?	“I sometimes or always experience difficulty in at least one area, even if…”	“No, but I use equipment, technology, assistance or other techniques to avoid difficulty”	No difficulty	Total
Yes	42	8	9	59
No	8	9	144	161
Total	50	17	153	220

**Table 6:** Results for questions D1 and D2 (Surveys 1 and 2).

	Survey 1	Survey 2
User friendliness of the question (how easy to understand) (Q6)	D1: 93.3%	D2: 90.9%
Further explanation of wording needed (Q7)	D1: 12.5%	D2: 15.5%
Which (question) do you feel more comfortable answering? (Q8)	D2: 52.1%, vs. D1: 47.9%	D1: 62.7%, vs. D2: 37.3%
Listing of everyday life areas with examples preferred (Q9)	67.7% yes, as in D1	69.1% yes, as in D1
Examples of “long-term health condition” helpful (Q10)	64.5% yes, as in D1	60.0% yes, as in D1
Whether report yes/no to “do you have a disability?” (Q11)	Yes: 16.6% No: 83.4	Yes: 26.8% No: 73.2

**Table 7:** Responses to environment questions.

	Survey 1	Survey 2
Answer to test question, i.e., need to improve/adjust aspects of environment (Q13)	**EN1—Improvement needed in** 23.0%—support and relationships 31.0%—attitudes 28.4%—services, systems, and policies 25.6%—products and technology 20.4%—natural environment and human-made changes 40.6%—no improvement needed	**EN2—Adjustments needed in** 16.4%—support and relationships 16.4%—attitudes 20.5%—services, systems, and policies 24.1%—products and technology 12.3%—natural environment and human-made changes 56.4%—no adjustment needed
How easy is this question to understand? (user friendliness) (Q14)	EN1: 5.1% rated EN1 as “not very easy to understand”	EN2: 1.4% rated EN2 as “very hard to understand, or not very easy to understand”
Further explanation of wording needed? (Q15)	Yes: 7.1% No: 92.9%	Yes: 11.4% No: 88.6%
Which question do you feel more comfortable answering? (Q16)	EN1: 58.8% EN2: 41.2%	EN1: 50.7% EN2: 49.3%
Improvements/adjustments to aspects of environment (Q18)	**EP1—Improvement made in** 14.4%—support and relationships 9.3%—attitudes 12.5%—services, systems, and policies 20.8%—products and technology 5.4%—natural environment and human-made changes 57.2%—no improvement made	**EP2—Adjustment made in** 11.6%—support and relationships 5.9%—attitudes 13.2%—services, systems, and policies 17.3%—products and technology 7.3%—natural environment and human-made changes 64.5%—no adjustment made
How easy is the question to understand? (user friendliness) (Q19)	EP1: 3.9% rated it as “very hard to understand, or not very easy to understand”	EP2: 3.4% rated it as “not very easy to understand”
Further explanation of wording needed? (Q20)	Yes: 6.0%	Yes: 10.3%
Which question do you feel more comfortable answering? (Q21)	EP1: 54.8% EP2: 45.2%	EP1: 52.0% EP2: 48.0%

## Supplementary Materials

The following are available online at https://www.mdpi.com/1660-4601/17/15/5435/s1, Supplementary S1: Questions for focus groups to discuss. Supplementary S2: Online survey questions. Table S1: Mapping 10 instruments to the ICF – overview. Table S2: Responses to disability questions. Table S3: Responses to environment questions. Table S4. Characteristics of Workforce Profile compared to People Matter Employee Survey.
